# miR-126 and miR-126*: New Players in Cancer

**DOI:** 10.1100/tsw.2010.198

**Published:** 2010-10-12

**Authors:** Jeannette Meister, Mirko H. H. Schmidt

**Affiliations:** Molecular Signal Transduction, Institute of Neurology (Edinger Institute), Johann Wolfgang Goethe University School of Medicine, Frankfurt am Main, Germany

**Keywords:** cancer, microRNA, epidermal growth factor–like domain 7, EGFL7, miR-126, miR-126*

## Abstract

Cancer progression is characterized by autarky in growth signals, insensitivity to growth-restrictive signals, evasion of apoptosis, a limitless potential to replicate, sustained angiogenesis, and tissue invasion, including metastasis. The regulation of these cellular processes relies on a fine-tuned control of molecular signal cascades. In recent years, short noncoding RNAs termed microRNAs (miRNAs) have been described as a novel class of molecular regulators. These affect various signaling cascades during the progression of neoplastic diseases by the regulation of gene expression on the post-transcriptional level. The novel endothelial cell–derived secreted protein epidermal growth factor–like domain 7 (EGFL7) has been suggested to control vascular tubulogenesis. Further, the two biologically active miRNAs miR-126 and its complement miR-126*, which are encoded by intron 7 of the egfl7 gene, have been described to mediate vascular functions. Knock-out studies in zebrafish and mice suggested a major role of miR-126 in angiogenesis and vascular integrity, which was mediated by the repression of inhibitors of VEGF-induced proliferation in endothelial cells. Recent studies revealed the distribution and function of miR-126 and miR-126* in various types of cancer, and assigned a role to both miRNAs as suppressors of tumor formation. Indeed, miR-126 and miR-126* have been reported to impair cancer progression through signaling pathways that control tumor cell proliferation, migration, invasion, and survival. Conversely, miR-126 and miR-126* may have a supportive role in the progression of cancer as well, which might be mediated by the promotion of blood vessel growth and inflammation. In this work, we will summarize the current knowledge on functions of miR-126/miR-126* that are relevant for cancer formation, and we will discuss their potential clinical use as predictive markers of survival and application as novel therapeutic targets for the treatment of neoplastic diseases.

## INTRODUCTION

In 1993, a small RNA molecule was discovered that controlled the developmental timing of the nematode *Caenorhabditis elegans*[1]. Since this initial discovery, the perception of RNA molecules has evolved.

Using computer-based predictions, numerous microRNAs (miRNAs) have been discovered and many of them have been proven to impact cell functions in most tissues, including various types of cancers. In general, miRNAs are considered to play a crucial role in proliferation, differentiation, development, apoptosis, and oncogenesis[2], suggesting that miRNAs are a novel class of cancer-relevant molecules. Intensive, comparative, microarray-based studies examined different patterns of miRNA expression throughout a variety of healthy tissues, tumors, and tumor-derived cell lines. In fact, specific miRNA expression profiles can be characteristic for certain types of tumors; therefore, specific miRNA signatures have been suggested to be relevant for the prognosis and prediction of cancer patient survival[3,4,5,6,7]. More recently, miRNAs have been detected in the peripheral blood[8], offering them as innovative biomarkers for early cancer detection.

One mechanism by which miRNAs may support tumor growth is the control of angiogenesis, the process of forming new blood vessels from pre-existing vessels. In order to uncover miRNAs as regulators of tumor angiogenesis *per se*, Lewis lung carcinoma cells have been implanted in mice lacking the endothelial Dicer protein, which is a terminal endonuclease crucial for the generation of miRNAs. Most interestingly, the lack of Dicer in endothelial cells resulted in a reduced rate of tumor neoangiogenesis[9].

One set of miRNAs important for endothelial cells are miR-126 and its complementary analog miR-126*, which both originate from a common precursor structure located within the *egfl7* gene. Both are highly expressed in the endothelium[10] and in hematopoietic stem cells[11]. In parallel, two publications reported severe defects in angiogenesis due to restricted VEGF signaling upon loss of miR-126 in zebrafish[12] and mice[10]. Accordingly, a role of miR-126/miR-126* in tumor neoangiogenesis seems possible. Changes in the expression levels of both miRNAs have been reported in various cancer cells originating from breast, lung, cervix, bladder, prostate, colon, liver, prostate, esophageal, or gastric cancer, as well as leukemia[3,4,7,13,14,15,16,17,18,19,20,21].

## BIOSYNTHESIS OF miRNAS

miRNAs are highly conserved, small, noncoding RNA molecules, 19–22 nucleotides in length, which control the expression of genes on the post-transcriptional level. Regulation is achieved by binding to the 3´ untranslated regions (3´ UTR) of cognate target messenger RNAs (mRNAs), which either leads to their degradation or the inhibition of mRNA translation (summarized in [22,23,24,25]). Most of the currently known miRNA sequences are located in introns and their expression is paralleled by the transcription of mRNAs encoded by the host genes. A lower percentage of miRNAs originates from exons or noncoding RNA-like regions[26]. Recently, intronic miRNAs have been reported to harbor regulatory elements upstream of the origin of transcription of their host genes. These elements displayed promoter characteristics and, accordingly, an independent expression of miRNAs and mRNAs has been suggested[27].

miRNAs, like conventional mRNAs, are transcribed by the RNA polymerase II (Pol II) complex[28] and are subsequently capped, polyadenylated, and spliced. Transcription results in a primary miRNA transcript (pri-miRNA) that harbors an indicative hairpin structure[29]. Within the nucleus, the RNAse III–type molecule Drosha and its cofactor DGCR8 process the pri-miRNAs into 70- to 100-nt-long pre-miRNA structures[30], which in turn are exported to the cytoplasm through the nuclear pores by Exportin-5[31]. Subsequently, the RNAse III–type protein Dicer generates a double-stranded short RNA in the cytoplasm[32] that consists of the leading-strand miRNA and its complementary miRNA sequence (referred to as miR/miR*). This duplex RNA is unwound by a helicase into a single-stranded miRNA and is assembled into the RNA-induced silencing complex (miRISC; Fig. 1)[33]. In most cases, one of the miRNAs is degraded[34]; however, in the case of miR-126/miR-126*, both miRNAs are stable and mediate characteristic functions.

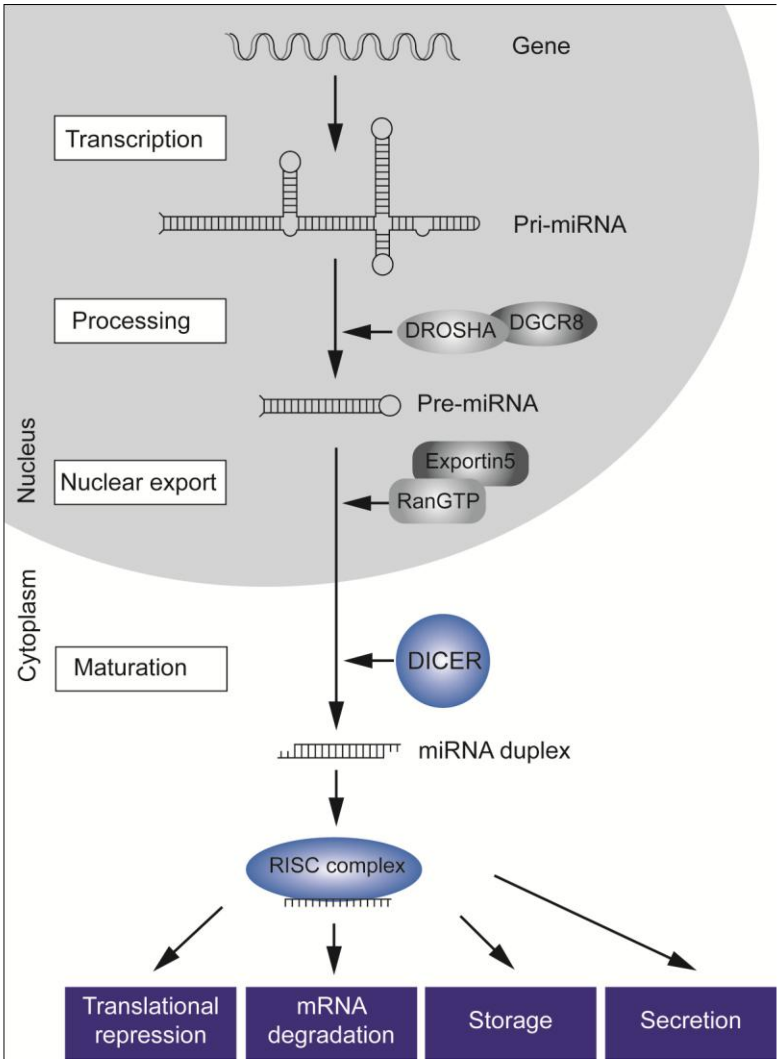

**FIGURE 1.** Biogenesis of miRNAs. miRNAs are transcribed as RNA precursor molecules (pre-miRNA), which are processed by Drosha and its cofactor DGCR8 into short hairpin structures (pri-miRNA). These are exported into the cytoplasm, where they are further processed into mature single-stranded miRNAs by the endonuclease Dicer. Those mature miRNAs are incorporated into the RISC complex and regulate post-transcriptional gene expression through various mechanisms.

## EGFL7 STRUCTURE AND EXPRESSION PATTERN

EGFL7 (synonyms: VE-statin, MEGF7, Notch4-like protein, Neu1, or Zneu1) has been described as an endothelial cell–derived, secreted inhibitor of smooth muscle cell migration and has been suggested as a regulator of blood vessel formation[35]. Further studies underpinned the significance of *egfl7* in the process of tube formation during vasculogenesis[36,37]. The EGFL7 protein is modularly assembled and contains an N-terminal signal secretion peptide (SS) followed by an Emilin-like domain (EMI), two epidermal growth factor–like (EGF) domains, and a coiled-coil (CC) region (Fig. 2)[38]. EGFL7 is highly expressed during embryonic development, but becomes down-regulated upon birth in the vascular system. In the adult vasculature, it remains restricted to sites of active vascular remodeling, e.g., during menstruation (physiological angiogenesis) and wound healing, tumor growth, or metastasis (pathological angiogenesis)[39]. Initial analyses reported EGFL7 expression to be restricted to the vascular endothelium

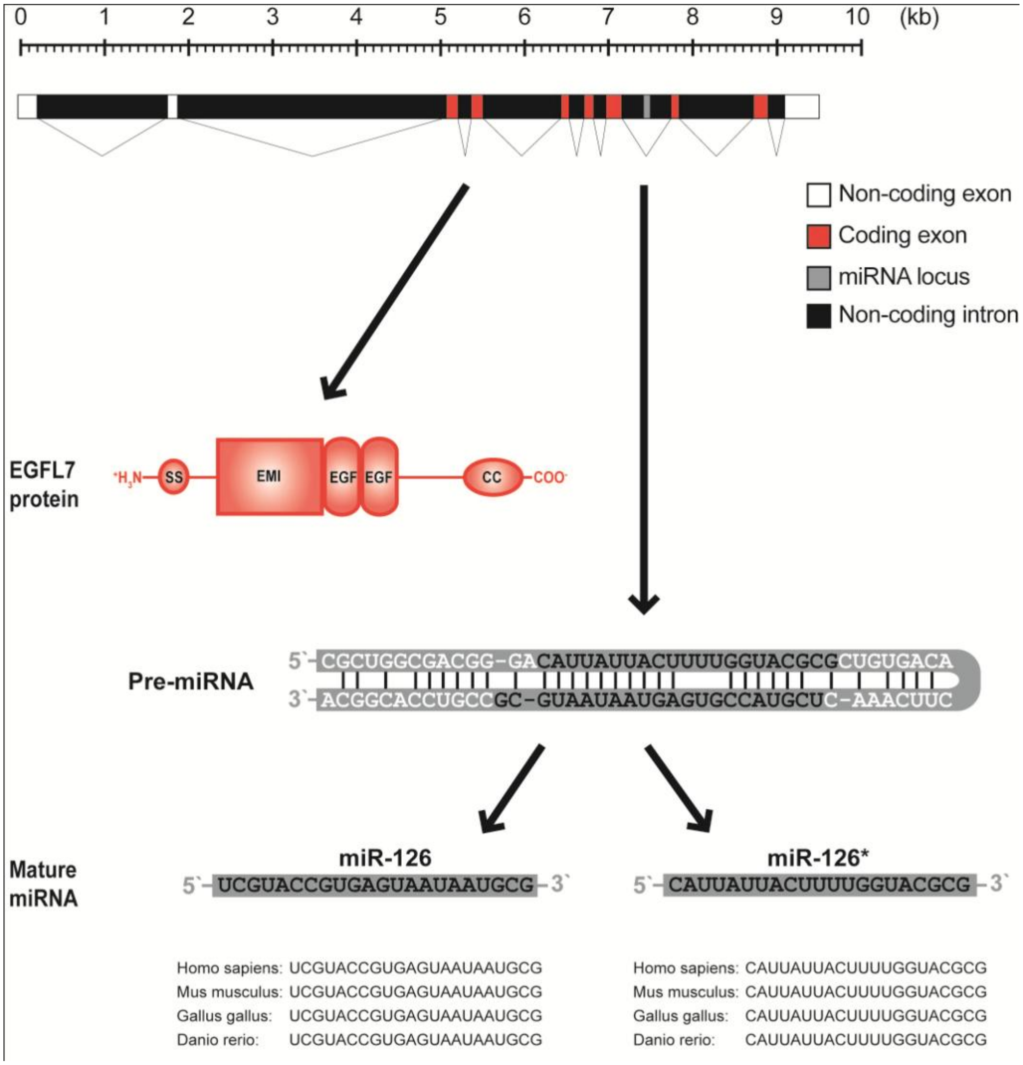

**FIGURE 2.** Structural organization and products of the *egfl7* gene. The *egfl7* gene consists of 10 exons, but only exons 3–9 encode for the EGFL7 protein, which displays a modular structure with an N-terminal signal secretion peptide (SS) followed by an Emilin-like domain (EMI), two epidermal growth factor–like domains (EGF), and a coiled-coil (CC) region. A pre-miRNA structure is located in intron 7 of the *egfl7* gene from which miR-126 and miR-126* originate. Both miRNAs have been highly conserved in evolution among different species.

at all stages of mouse development[36,40], however, subsequent analyses implicated EGFL7 in the primordial germ cell differentiation[39]. Additionally, EGFL7 expression has been observed in adult mice in a small subset of vessels in highly vascularized organs, such as the lung, heart, uterus, ovary, and kidney[35], as well as in neurons[41,42].

## miR-126 AND miR-126*

miR-126 (also referred to as miR-126-3p) and its complement miR-126* (miR-126-5p or miR-123) are derived of the *egfl7* gene, which harbors both miRNAs within intron 7 (Fig. 2) in all vertebrates[10,12]. In 2002, Lagos-Quintana et al. identified miR-123 and miR-126 in a tissue-specific mouse screen[43], which were later found to originate from the same precursor; therefore, miR-123 was renamed miR-126*[44]. Both miRNAs are conserved among species (http://microrna.sanger.ac.uk/sequences/index.shtml). Expression analyses revealed an abundant level of miR-126 in highly vascularized tissues, and identified miR-126 as the only miRNA known to be specifically expressed in the endothelial cell lineage, hematopoietic progenitor cells, and endothelial cell lines[10,12,18,44,45,46]. Unfortunately, only little specific information on miR-126* is available. Most information is derived from the knowledge on its complement miRNA miR-126, which is expressed in highly vascularized tissues such as heart[47], liver[43,44], or lung[44,48], or in human umbilical vein endothelial cells (HUVEC)[18]. In general, the expression of miR-126/miR-126* seems to be paralleled by the expression of the EGFL7 protein, as they share a common mRNA[12,35]. Nevertheless, there are studies that reported that miR-126 expression was regulated independently of the EGFL7 protein[49], suggesting the existence of a separate promoter that drives the expression of miR-126/miR-126*[27]. However, this interesting possibility needs to be challenged by further studies. Considering their expression pattern, miR-126/miR-126* have been attributed various roles in developmental angiogenesis, but also in carcinogenesis and inflammation.

## miR-126/miR-126* IN CANCER

During recent years, transcriptional profiling and microarray-based analyses revealed numerous miRNAs that display significant differences in their total expression levels comparing tumor and corresponding noncancerous tissues. One of these, miR-126, was reported to be lost in human breast cancer. Retroviral restoration of miR-126 levels in primary breast cancer cells suppressed the overall tumor growth and metastasis to the bone and lung in nude mice[7]. Notably, patients with primary breast tumors characterized by low miR-126 expression displayed very poor overall metastasis-free survival as compared to patients with tumors that exhibited high miR-126 levels. This finding suggests a clinical correlation between the loss of miR-126 and metastasis-free survival[7]. Consistently, down-regulation of miR-126 in cancerous vs. noncancerous tissues has been reported for cancers of the lung[50,51], stomach[21], cervix[14], bladder, and prostate[20]. Likewise, loss of miR-126* has been reported for colon[52], lung[50], and prostate cancer cell lines[18]. However, the functions of miR-126/miR-126* in these types of cancer appear to be diverse and largely unknown. Liu et al. reported VEGF-A to be down-regulated by miR-126, as the infection of lung carcinoma cell lines with lentiviral miR-126 caused a decrease in VEGF-A. This resulted in a cell cycle arrest of lung cancer cells and the inhibition of tumor cell growth *in vitro* and *in vivo*[47]. Further, Li et al. demonstrated that the transformation of mouse embryonic Cx43KO brain cells with the oncogene v-Src caused a decrease in miR-126 and miR-126* expression. Treatment of breast cancer cell lines with the Src kinase inhibitor PP2 resulted in an increase of miR-126 expression, leading to decreased cell migration[49]. Additionally, the 3´ UTR of Crk, a component of the focal adhesion network involved in integrin signaling, has been reported as a target of miR-126. Interestingly, decreased Crk expression has been shown to suppress tumor cell proliferation and invasion[49,53]. In human colon cancer, Guo et al. suggested miR-126 as a tumor suppressor, and reported an association between the loss of miR-126 and an impaired signaling via phosphatidylinositol 3-kinase (PI3K) due to the down-regulation of its regulatory subunit p85β[52]. Recently, miR-126 has been suggested as a tumor suppressor in human gastric cancer since miR-126 inhibited tumor growth and metastasis *in vivo* and *in vitro*. Partially, this effect was mediated by down-regulation of Crk[21]. In sum, various molecular signal cascades involved in the regulation of cancer cells have been described to be modulated by miR-126/miR-126* and *vice versa* in a cell type–specific and context-dependent manner (Fig. 3).

## miR-126/miR-126* IN ANGIOGENESIS

The functions of miR-126/miR-126* in angiogenesis remained enigmatic for quite some time. Recently, Nicoli et al. demonstrated the induction of miR-126 expression by blood flow, which was mediated by the mechanosensitive zinc finger–containing transcription factor *klf2a* and resulted in the activation of VEGF

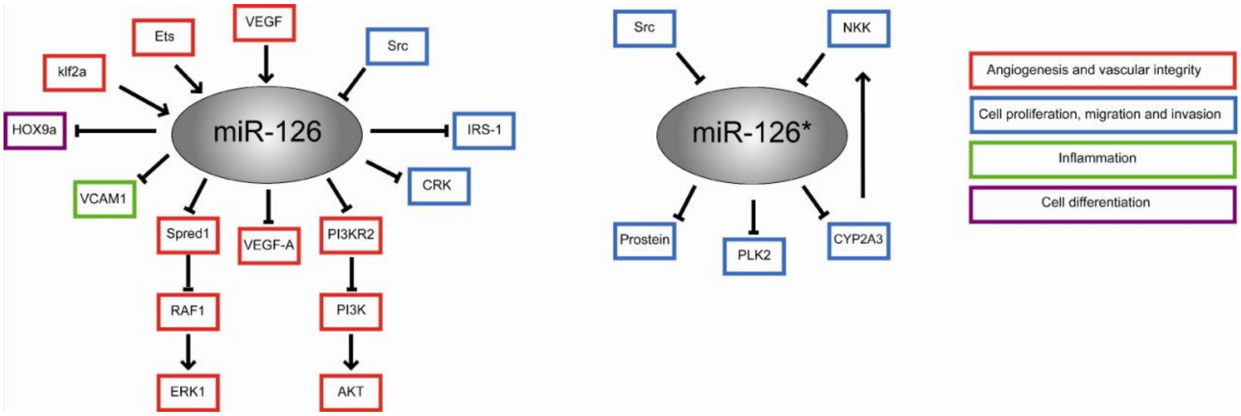

**FIGURE 3.** Schematic of the signal pathways and cell functions affected by miR-126/miR-126*. Multiple factors control the expression of miR-126/miR-126* during the processes of angiogenesis and cancer progression. In turn, expression of miR-126 results in a blockade of signaling pathways that govern angiogenesis, vascular integrity, inflammation, and differentiation. Additionally, miR-126 and miR-126* may act as tumor suppressors via the inhibition of proliferation, migration, and invasion of cancer cells.

signaling in the endothelium[54]. Further, miR-126 knock-down studies in zebrafish[55] and mice[10,56] led to impaired endothelial cell migration during vessel growth, collapsed vessel lumens, and a compromised endothelial tube organization *in vivo*[38]. Partially, miR-126 mediated its effects by the restriction of VEGF-induced signals, as it repressed Sprouty-related EVH1 domain–containing protein 1 (SPRED1) and a subunit of PI3K (PI3KR2, synonym: p85β). Both proteins inhibit receptor tyrosine kinase–induced signaling via the MAPK and PI3K pathways[10,12,52]. These observations are paralleled by studies that demonstrated the binding of miR-126 to the 3´ UTR of the VEGF-A mRNA. Down-regulation of VEGF-A was accompanied by a cell cycle arrest of the three lung cancer cell lines – A549, Y-90, and SPC-A1 – in the G1-phase and its reduced tumor growth in nude mice[47]. In sum, miR-126/miR-126* both promote the development of the cardiovascular system and their functions have been intensively studied in the formation of vascular disorders, such as abnormal angiogenesis and vascular leakage[10] and in various types of neoplastic diseases.

## miR-126/miR-126* AND INFLAMMATION

Epidemiological evidence indicates that up to 25% of all cancers are induced by infectious processes[57]. During inflammation, the expression pattern of oncogenes and tumor-suppressor genes, as well as noncoding miRNAs, dramatically changes. Indeed, miR-146a and b have both been shown to hybridize with sequences in the 3´ UTRs of the TNF receptor–associated factor 6 and IL-1 receptor–associated kinase 1 genes, thereby decreasing two key adapter molecules downstream of Toll-like and cytokine receptors[58]. Recently, two publications on miR-126 implicated a role of miRNAs in inflammation. First, miR-126 has been shown to inhibit the expression of the vascular cell adhesion molecule (VCAM1) in endothelial cells, thereby blocking adhesion and infiltration of leukocytes into the vasculature wall. In addition, endothelial cells transfected with an antisense construct to target endogenous miR-126 expression failed to respond to stimulation of the endothelium with the cytokine TNF-α to promote VCAM1 expression[45]. Second, Wu et al. reported an altered expression of miR-126 in ulcerative colitis and suggested a role for miR-126 in the development of colon cancer[59]. However, the differential expression of miRNAs and their role in inflammation have only started to be elucidated.

## CONCLUDING REMARKS AND FUTURE PERSPECTIVES

A growing body of evidence indicates that miRNAs convey a novel and efficient way for the regulation of gene expression, thus being involved in multiple aspects of cellular processes. Especially, miR-126 and miR-126* have been intensively studied and have been attributed significant roles in different physiological and pathological processes, including inflammation[45], blood vessel growth[10], and cancer[7].

As discussed in this review, miR-126/miR-126* impair cancer progression through various pathways. It has been shown that miR-126/miR-126* inhibited tumor growth by the prevention of cancer cell proliferation, migration, and invasion, but also by down-regulation of central signal molecules of cancer cell survival. The tumor-suppressive functions of miR-126/miR-126* have been studied in different types of neoplastic diseases. However, it is difficult to connect the functions of miR-126/miR-126* in cancer cells to the physiological functions of both miRNAs in the vasculature. In fact, it has been described in several animal models that miR-126 is essential for endothelial cells to respond to growth factors[10,12,54], thus acting as a promoting factor for endothelial cell proliferation and migration, which is counterintuitive for the actions of a putative tumor suppressor.

Unfortunately, there is only little knowledge on the role of miR-126/miR-126* in tumor neoangiogenesis. It has been well established that blood vessel growth represents one of the key features in the pathogenesis of cancer[60]; therefore, it is of great interest to compare the effects of miR-126 in tumor cells to the functions of miR-126 in the tumor vasculature and in the surrounding vessels of the tumor periphery. In case miR-126/miR-126* is up-regulated in the tumor vasculature during tumor progression, this might promote vessel neoangiogenesis to support tumor growth and metastasis; *vice versa*, if it is down-regulated in tumor vessels, this might lead to defects in the vascular integrity, which could partially explain abnormal tumor vessel phenotypes (Fig. 4). However, these actions remain highly speculative, since there are no current studies published on the role of miR-126/miR-126* in tumor neoangiogenesis.

Another interesting question arises from the functional differences between miR-126 and miR-126*. During miRNA maturation, one of the strands of the miR-126/126*-heterodimer is likely to be degraded[22]; however, the mechanism to choose the “right” miRNA for degradation remains unclear. In the case of miR-126/miR-126*, both miRNAs have been detected within the same tissue[20]. In contrast, the question if miR-126 and miR-126* are expressed within the same cell lineage or even within one cell remains to be investigated. In theory, the two miRNAs should be expressed in a mutually exclusive manner because each represents its own antisense sequence and should therefore be capable of inhibiting itself. However, there are examples of miRNAs encoded by a common precursor carrying out distinct biological functions, but being expressed in a tissue-specific manner. As an example, miR-1 and miR-133 share the same precursor, but are collectively expressed in the muscle. Interestingly, they perform opposing roles in skeletal muscle proliferation and migration. miR-1 promotes myogenesis by targeting histone deacetylase 4 (HDAC4), a transcriptional repressor of muscle gene expression. In contrast, miR-133 enhances myoblast proliferation by repression of serum response factor (SRF)[61].

The tumor-suppressive functions of miR-126/miR-126* offer these miRNAs as novel cancer therapeutics. Several studies identified miR-126 as a novel prognostic marker to predict the overall survival rate of patients in some types of cancer. Tavazoie et al. reported a clinical association between metastasis-free survival and high levels of miR-126 expression in breast cancer[7]. In addition, miR-126/miR-126* levels are down-regulated in cancers derived of lung[50,51], stomach[21], cervix[14], bladder, and prostate[20], maybe due to tumor-suppressive effects. In conclusion, the restoration of endogenous miR-126/miR-126* levels might help to directly target distinct pathways that promote cancer progression. Currently, inhibitors of the VEGF signaling pathway, like bevacizumab, sunitinib, and sorafenib, are clinically tested, but therapeutic benefits are often transient and followed by a rapid restoration of tumor growth and progression[62]. In this context, the restoration of endogenous miR-126/miR-126* levels might be an alternative to monoclonal antibody–based anti-VEGF therapies.

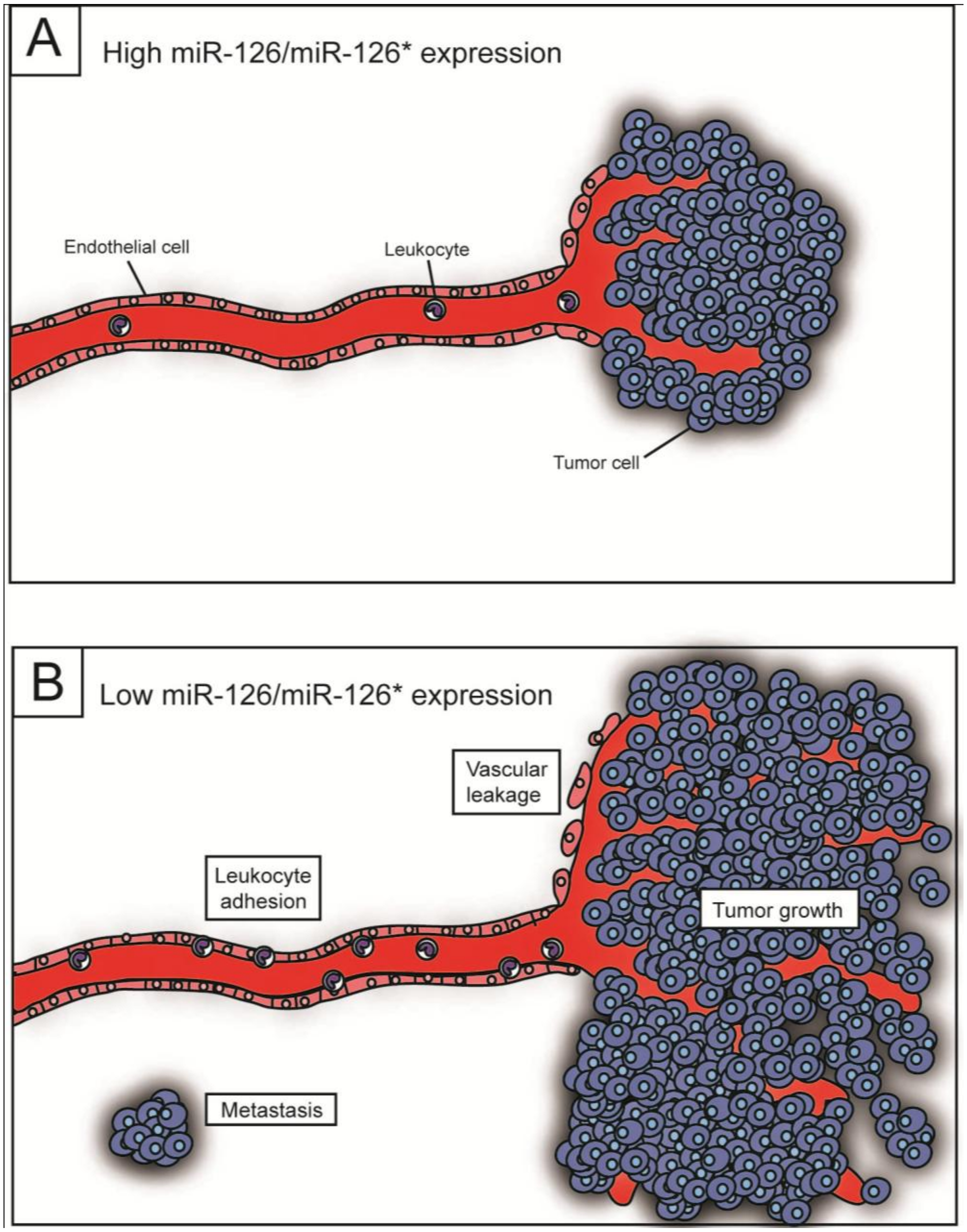

**FIGURE 4.** Putative effects of miR-126/miR-126* on the progression of cancer. (A) High levels of miR-126/miR-126* drive proper vessel formation by modulation of the response of endothelial cells to vascular growth factors and restriction of leukocyte adhesion. (B) Loss of the tumor-suppressor miR-126/miR-126* during cancer progression may result in elevated tumor cell proliferation, migration, and survival; enhanced formation of metastasis; and an increase in leukocyte adhesion followed by their infiltration into the vascular wall. Further, loss of miR-126/miR-126* might disturb vascular integrity, leading to a disorganized and abnormal tumor vasculature.

This raises the question of the benefits of miRNA treatment over existing therapeutic approaches. First, individual miRNAs are able to control gene expression networks; therefore, many cellular pathways are simultaneously targeted in a specific manner, and tumor cell escape and reappearance could be less likely. Second, miRNA restoration mimics physiological conditions and side effects might be less pronounced. Despite that certain miRNAs are overexpressed in cancer cells, the vast majority seems to be down-regulated in tumors[6]. Global miRNA repression enhances cellular transformation and tumorigenesis *in vitro* and *in vivo*[63]. Currently, various methods of miRNA delivery, like viral transfer, transfection of cells with miRNA-encoding plasmids, or the direct delivery of oligonucleotides to cells, are under development[64]. As a proof of principle, therapeutic restoration of miR-26a has been shown to suppress tumorigenesis in a murine liver cancer model *in vivo*[65].

In conclusion, the role of miR-126/miR-126* in the complex process of cancer formation remains largely unknown at the moment. In the future, it will be very interesting to study the role of both miRNAs in cancer development, and to analyze how they affect tumor cells themselves and the blood vessels these cells attract. This might lead to the development of novel tools that simultaneously inhibit tumor cell growth and stimulate the maturation of blood vessels. Such compounds might prove to be powerful weapons in the war on cancer.

## ACKNOWLEDGMENTS

This work was financially supported by the German Research Foundation DFG within the framework of the Transregional Collaborative Research Centre 23 (subproject A4) and the Excellence Cluster 147 “Cardio-Pulmonary Systems”.
